# A survey on opinions, perceptions and attitudes of Swedish veterinarians—the Swedish veterinary disciplinary board

**DOI:** 10.3389/fvets.2025.1732118

**Published:** 2026-01-08

**Authors:** Agneta Egenvall, Karolina Brunius Enlund, Terese Holmquist, Magnus Rosenquist

**Affiliations:** 1 Department of Clinical Sciences, Faculty of Veterinary Medicine and Animal Husbandry, Swedish University of Agricultural Sciences, Uppsala, Sweden; 2 Holmquist Juridik AB, Täby, Sweden; 3 Swedish Veterinary Association, Stockholm, Sweden

**Keywords:** complaint, regulation, sanction, veterinarian, veterinary disciplinary board

## Abstract

**Introduction:**

Veterinary disciplinary boards (VDBs) evaluate complaints from animal owners regarding the healthcare of animals. It is important that these boards safeguard the quality of veterinary care, but how veterinarians are affected psychosocially is also relevant. The aim of this study was to explore perceptions, attitudes and experiences among veterinarians concerning the Swedish VDB, taking account of respondent characteristics.

**Materials and methods:**

A web-based questionnaire targeting veterinarians in current or former clinical practice was launched in 2024. There were 1,054 responses initiated and of these 819 were completed.

**Results:**

Half of the respondents had had complaints filed against them, but only 1 in 10 had received a sanction. Few veterinarians left clinical work because of VDB-related issues. Many veterinarians were relatively unaware about VDB processes and did not worry excessively about complaints being filed, respondents replying ‘cannot evaluate’ ranged 33–61% for these questions. However, 18% worried very much about having complaints filed. While veterinarians identified that VDB assessments increased record keeping and emphasis on communication, they generally felt that the VDB had relatively little effect on the patient outcomes for animals seen. Some animal owners who had previously submitted complaints would be denied access if veterinarians were aware of those complaints. Instituting operational oversight was considered a valuable adjunct to current regulation (43.7% ‘agreed completely’).

**Discussion:**

Overall, respondents found that the VDB performs an important task, while areas for improvement were identified, including transparency of procedures. One way to mitigate worries of complaints could be increased education and training in soft skills, including skills for communication and management of complaints.

## Introduction

An overarching aim with veterinary disciplinary boards (VDBs) is to secure quality of care. This is usually done by evaluating complaints from clients or regulatory bodies (1). Although systems vary widely, many VDBs may impose sanctions that can directly or indirectly lead to withdrawal of a licence to practice veterinary medicine. Research on VDBs include questionnaire studies targeting veterinarians’ experiences and attitudes to VDBs in the US (2) and the Netherlands (3). Actual complaints from the Netherlands (1) and the US (4, 5) have been studied to understand reasons and determinants of these. Similarly, complaints from New Zealand were studied with thematic analysis (6). Complaints from Sweden have also been studied with thematic analysis (7), and also specifically complaints related to euthanasia (8). Common reasons attributed to submitted claims in these studies, except for medical errors, were lack of communication and inadequate clinical record keeping. One qualitative study from the UK found that complaints filed against veterinarians had profound negative effects both on the veterinarians and on their work (9). Also others (5), categorised sanctioned complaints in California and found that inadequate record keeping was a strong determinant for receiving sanctions. In human medicine, the issue of disciplinary boards and its effects on the care and the caregivers have been studied widely (10).

The Swedish VDB (Ansvarsnämnden för djurens hälso- och sjukvård, the Swedish Veterinary Disciplinary Board (11)) is a governmental authority that examines cases concerning disciplinary measures against veterinarians and other licensed animal health personnel (12). The purpose of the VDB is to ensure high standards of animal health care, maintain public trust in the profession, and safeguard legal certainty in the handling of cases. The Swedish VDB mainly scrutinises complaints from animal owners regarding alleged inadequate veterinary care by veterinarians and other licensed animal health personnel that has occurred during treatment of their animals (11). This involves veterinary procedures that a person belonging to the animal health personnel have used, or not used, to make a diagnosis, examine or treat animals. This also involves certificates (e.g., certificate of absence of a certain disease condition or for insurance eligibility) and record keeping, but it does not involve care costs or personal conduct. A complaint to the VDB may be filed by the animal owner or another animal keeper, a person otherwise responsible for the animal (e.g., a caretaker), or the County Administrative Board, in its role as supervisory authority (12). It is not possible for members of the public in general, or for other veterinarians, to submit a complaint unless they fall within one of the categories above. If the VDB finds that a veterinarian or other licensed animal health personnel have breached their professional obligations, the Board may impose a sanction. The sanctions available (12) are: a reprimand (in Swedish ‘erinran’, less severe) and a warning (Swedish ‘varning’, more severe). The VDB may also dismiss a complaint if it is manifestly unfounded or outside its jurisdiction, e.g., only relates to cost of care. Evaluated complaints resulting in sanctions are passed on to the Board of Agriculture, which assesses whether further action is needed, even though the VDB makes its decisions independently of the Board (11). The VDB may revoke the license to practice as a veterinarian if they determine that the reported veterinarian has accumulated enough severe complaints or acted with gross negligence (12). A complaint to the VDB must (12), be filed within 2 years from the event to which it relates. Incidents occurring more than 2 years prior to filing will not be considered. The handling time varies, but according to the VDB’s own information, the process often takes about 1 year (11).

Through a parliamentary decision (13), based on a government report concerning sustainable, well-functioning and long term animal health care sector in Sweden (14), a new supervisory function referred to as operational oversight has been introduced. This body will handle complaints related to organisational deficiencies and working environment factors beyond the veterinarian’s control. The aim is to relieve individual veterinarians of responsibility for matters that are in fact the employer’s responsibility, such as staff shortages, inadequate equipment, or substandard facilities. This update of organisation responsibility is thereby anticipated to relieve stress from veterinarians.

The system was/is considered important for maintaining quality in veterinary care. However, recent studies suggest that the number of complaints as well as the stress that is caused by the disciplinary procedures, may become counter-productive as far as quality of veterinary care is concerned (2, 3). Veterinarians have high suicidal rates and problems with mental health (15, 16). Compassionate personality types of veterinarians, high pressure from clients, high care costs (17) and seeing animal owners forced to choose euthanasia instead of adequate care, because of costs, are among suggested reasons (15, 18). While reasons for increased levels and costs of care likely are many, VDBs may also drive standards and prices. This is especially true if veterinarians practice ‘defensive medicine’ in order to avoid complaints, i.e., do more extensive investigations because of fear of complaints or legal actions (19). To do more examinations than needed can be contrasted to ‘spectrum of care’ (20, 21)/‘contextualised care’ (22). These latter concepts entail, while not compromising animal welfare, tailoring examinations and treatment while also taking into consideration the needs of the animal owner, even if these are not gold standard. Effects on the veterinary profession from VDBs tie into studies of the general health of veterinarians.

VDBs have been studied in different countries, during different times and with various methods and objectives, with both different and similar effects on veterinary populations having been reported. Given that a new addition to the oversight had recently been decided in Sweden it was deemed useful to address the current perceptions and experiences of veterinarians in Sweden of the current VDB and opinions of the new addition. This study provides a baseline that allows for future comparison to the new oversight system, as well as international comparisons and insight into the effects of the VDB on Swedish veterinarians.

In the current study, we hypothesised that Swedish veterinarians are mainly negatively affected by the VDB, both when having complaints filed and generally in their work affecting the veterinary care delivered. The aim was to explore opinions, perceptions, attitudes and experiences among veterinarians concerning the Swedish VDB, taking account of respondent characteristics.

## Materials and methods

### Questionnaire design

A web-based questionnaire (Supplementary Table 1 sheet Overall) was designed using Netigate (23). The cross-sectional survey targeted mainly veterinarians in current or former clinical practice. Before launching it was pretested with four individuals and edited. Human ethical permit was not necessary according to rules of the Swedish Ethical Review Authority (24), as the questionnaire was anonymous and contained no sensitive personal data. Questions were posed in Swedish, and the questions have been translated for this presentation (Supplementary Table 1). Respondents gave consent to being included by answering the questionnaire.

We designed questions through discussion within, and outside of, the research team. The questions were mainly closed with Likert-scale response options, along with seven open-ended questions allowing respondents to elaborate on their answers. Free-text responses are not used in the current presentation. The option ‘cannot evaluate’ was also provided. The questionnaire was organised into 11 areas (Supplementary Table 1). One area (no 9) did not cover questions relating to the VDB and will be presented separately (whether veterinarians report animal owners to the County Administrative Board).

- Respondent background information (area 1).
- If the respondent had changed from clinical work (area 2).
- Workplace information (area 3).
- If the respondent was aware of the case verdicts from the VDB, and perceptions on how the VDB executes its task (area 4).
- If verdicts of cases evaluated by the VDB affects respondents’ work (area 5).
- Whether animal owners previously filing complaints or creating other problems would be received at the clinic (area 6).
- To respondents that had complaints filed to the VDB, how support was received and sought (from the veterinary community as well as specific advice, area 7a).
- How experiences of having complaints filed has affected work routines (area 7b).
- Perceived reasons for complaints filed to the VDB (area 8).
- Perceptions on the newly decided operational oversight and opinions about how an ‘ideal’ VDB could be constructed (area 10).

These areas were selected based on providing background information (areas 1–3), general questions on how veterinarians both with and without previous sanctions experience the VDB (areas 4–5) and questions on how veterinarians would receive owners with previous complaints filed (area 6). The next area was related specifically to veterinarians having had complaints filed (area 7). Then we wanted to gain information on what was perceived to lead to complaints (area 8) and finally question on how a well-working supervision could work (area 10).

### Distribution

The questionnaire was open from 11 December 2024 to 15 February 2025. The link to the questionnaire, together with an invitation to participate, was distributed through emails to members in The Swedish Veterinary Association (11 December 2024, 2,400 active members) and through the Swedish private Facebook sites ‘peptalk för veterinärer’ (7 January 2025, 3,400 members), ‘veterinärmedicin stordjur’ (10 January 2025, 2,500 members) and ‘veterinärmedicin smådjur’ (31 January 2025, 3,000 members). A reminder was sent out to the members on 17 January 2025.

### Data handling

Data were downloaded via the Netigate website as Excel files and processed in Matlab (version R2024b, Mathworks, Natick, MA, USA) using custom-written scripts. For most questions all answers were included, also from respondents not completing the questionnaire (note that a completed questionnaire did not necessarily contain answers to all questions, as responses were not obligatory). Exceptions from this was when questions were only relevant to subsets of the population. To evaluate the impact of including partial responses, percentages were compared between categories for all and answers from completed questionnaires (where the respondent finished the questionnaire by clicking the ending option). Supplementary Table 1 (sheet overall) shows that for only 6.3% of 379 analysed categories, the responses differed more than 1 percentage, indicating that this decision had a minor impact on the results. This indicates that the proportions for all alternatives were similar in the whole sample and the subsample with the completed answers.

### Statistical analysis

All variables were categorical or from Likert scales, in both cases treated as categorical. For evaluating whether all response alternatives were equally common or not, 95% binomial confidence intervals (95% CIs) were calculated. Alternatives such as ‘cannot evaluate’ or ‘do not know’ have been included in the statistics.

Questions on perceptions, opinions, experiences and knowledge about the VDB were tabulated against gender, duration of work experience, whether having left clinical work, size/organisation of workplace, whether having complaints filed (Supplementary Table 1) and for those with complaints filed were acquitted, versus given a warning or a reprimand.

## Results

### General results

There were 1,054 respondents initiating the questionnaire (answering at least one question) and 819 respondents had clicked to end the questionnaire, i.e., the questionnaire was ‘completed’. For the completed answers the time to answer ranged from 2 min and 17 s up to 140 min and 11 s. The median response duration was 17 min and 34 s. All answers when provided were used, if not otherwise stated (see Supplementary Table 1). To minimize drop-out, answers were not obligatory for any question. Hence, there were different numbers of responses for different questions. Also, to aid for respondents the alternative ‘cannot evaluate’ was frequently introduced into the questions. Supplementary Table 1 shows total results, both with and without stratification by several variables (e.g., gender, workplace type, complaints filed, switched to non-clinical work), including graphs for each question for the stratified responses.

### Respondents (area 1)

Most respondents were women (82.3%, Table 1). Most respondents had worked for over 20 years in the veterinary clinical profession (36.4%), followed by 11–20 years (31.2%) and 1–10 years (30.7%). Within the group of women, those with 1–10 years, 11–20 years and over 20 years of experience were represented by equal shares (32.1–33.6%). Of all men, 58.0% had worked >20 years, 24.4% 11–20 years and 16.5% for 1–10 years. Most respondents were educated in Sweden (73.5%) (Table 1). Of the 1,034 respondents that provided at least one species common in their practice, 84% of the respondents attended dogs, followed by cats (82%), horses (35%), small mammals (26%), large ruminants (21%), small ruminants (14%), ‘caged’ birds (4%) and reptiles (2%).

**Table 1:** Respondent information (areas 1–3) from a questionnaire concerning the Swedish Veterinary Disciplinary Board distributed to veterinarians working in Sweden during winter 2024/2025.

Question/category	*n*	%	95% CI
Gender:	1,054		
Other/will not specify	8	0.8	(0.3, 01.5)
Woman	867	82.3	(79.8, 84.5)
Man	179	17.0	(14.8, 19.4)
Number of years with clinical experience as a veterinarian:	1,034		
>1 year	18	1.7	(1.0, 02.7)
1–10 years	317	30.7	(27.9, 33.6)
11–20 years	323	31.2	(28.4, 34.2)
>20 years	376	36.4	(33.4, 39.4)
Did you receive your veterinary education in Sweden?	910		
Yes	669	73.5	(70.5, 76.4)
No	241	26.5	(23.6, 29.5)
Have you switched to a primarily non-clinical service?	1,018		
Yes	175	17.2	(14.9, 19.7)
No, continue to the next page	843	82.8	(80.3, 85.1)
Are you employed by a company that owns more than 5 clinics?	850		
Yes	380	38.5	(35.5, 41.7)
No	470	47.7	(44.5, 50.8)
Government employee or district veterinary officer	136	13.8	(11.7, 16.1)
Do you own (with or without another person/persons) the clinic where you work?	930		
Yes	240	25.8	(23.0, 28.7)
No	690	74.2	(71.3, 77.0)
The size of the clinic (workplace) where you work—number of full-time positions (all staff categories)	927		
1–3	185	20.0	(17.4, 22.7)
4–8	167	18.0	(15.6, 20.6)
>8	575	62.0	(58.8, 65.2)

Presented are numbers in column 2, percentages and 95% confidence intervals (95% CI).

### Respondent having changed from clinical work (area 2)

**Figure 1:**
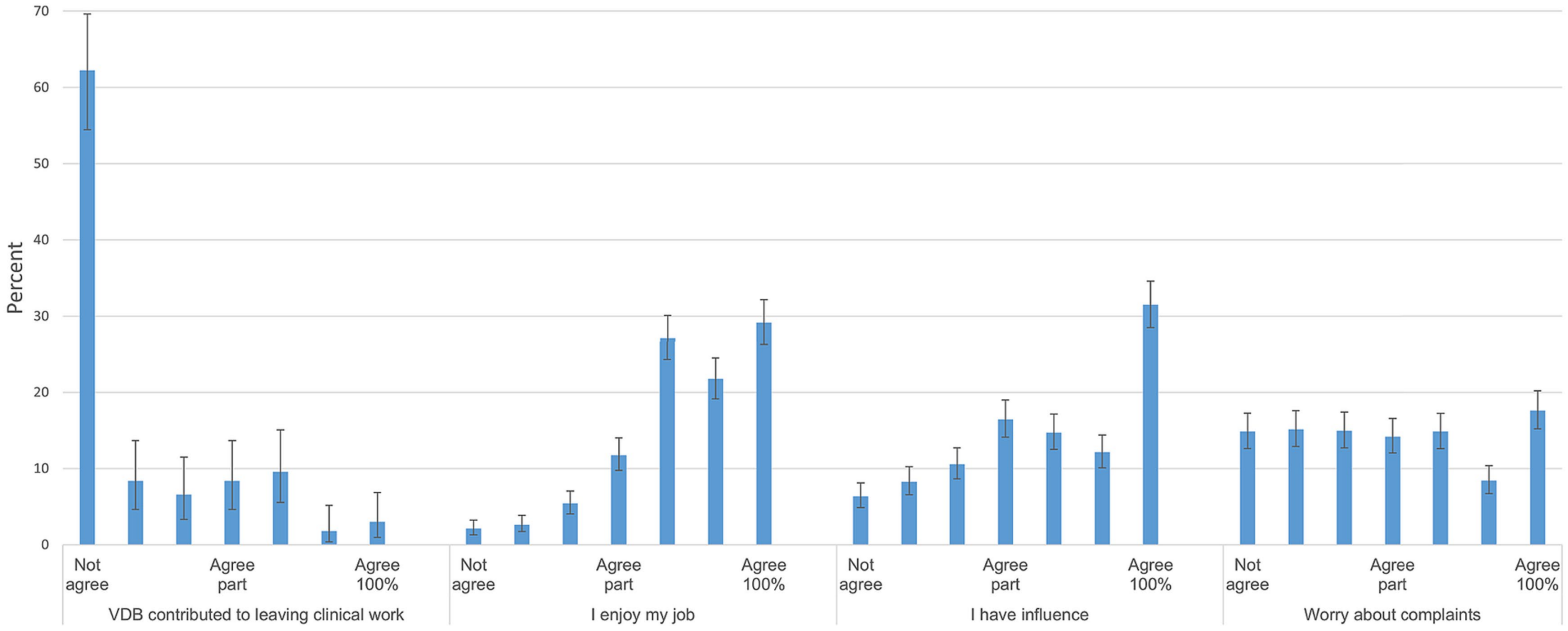
Working environment. Response distributions to Likert-scale questions from Area 2: If you earlier worked in clinical practice, but later changed to other work and Area 3: Workplace and working environment. The alternatives include a 7-step scale from does not agree (not agree) to agrees completely (agree 100%). Denominators from left to right are 167, 943, 930 and 937. 95% confidence intervals are included. Questions in full were posed as: ‘When the choice was made to change from clinical work, did you find that the Swedish Veterinary Disciplinary Board (VDB) or the current regulatory work, contributed to this decision?’, ‘I enjoy my job’, ‘I have influence over issues that interests me, e.g., treatment regimens, scheduling or pricing of veterinary care’, ‘I worry about having complaints filed to the VDB’.

Of 1,018 respondent 17.2% had left clinical work. In most cases, 62.3% (of *n* = 167) the VDB had no influence on the decision to leave clinical work, while for the rest of respondents (37.7% of the ones leaving and 6.2% of all [*n* = 1,018]) the VDB was considered to have had small or some impact (Figure 1).

### Workplace information (area 3)

Most respondents (47.7%) worked in enterprises that owned a maximum of 5 clinics (Table 1), 38.5% in enterprises owning more than 5 clinics and 13.8% were hired by the state or were district veterinary officers (25). In total, 74.2% stated they did not own their workplace enterprise and 25.8% that they did. In 62.0% the workplace was staffed by more than 8, in 20.0% by 1–3 and in 18.0% by 4–8 persons. Regarding enjoying work, 10.2% stated that they did not enjoy work (combing the 3 first categories) (Figure 1). Combining the first 3 categories for having influence on questions of interest, 25.2% felt they did not have influence. The different seven alternatives available for whether worrying about having complaints filed were evenly filled out, with 40.9% tending to worry (combining the last 3 categories) (Figure 1). The answers regarding having changed from clinical work were tabulated against type of workplace (972 respondents, Supplementary Table 1- sheet LeftVsWorkPlace). It was found that those affiliated with DV had changed from clinical work to a much high degree than those in working in small or larger companies (DV 38.5% [95% CI; 30.3–47.3], clinics owned by companies owning >5 clinics 9.7% [95% CI; 6.9–13.1] and smaller clinics 14.2% [95% CI; 11.2–17.7]).

Following cases from the VDB, and perceptions about the processes at the VDB (area 4).

**Figure 2:**
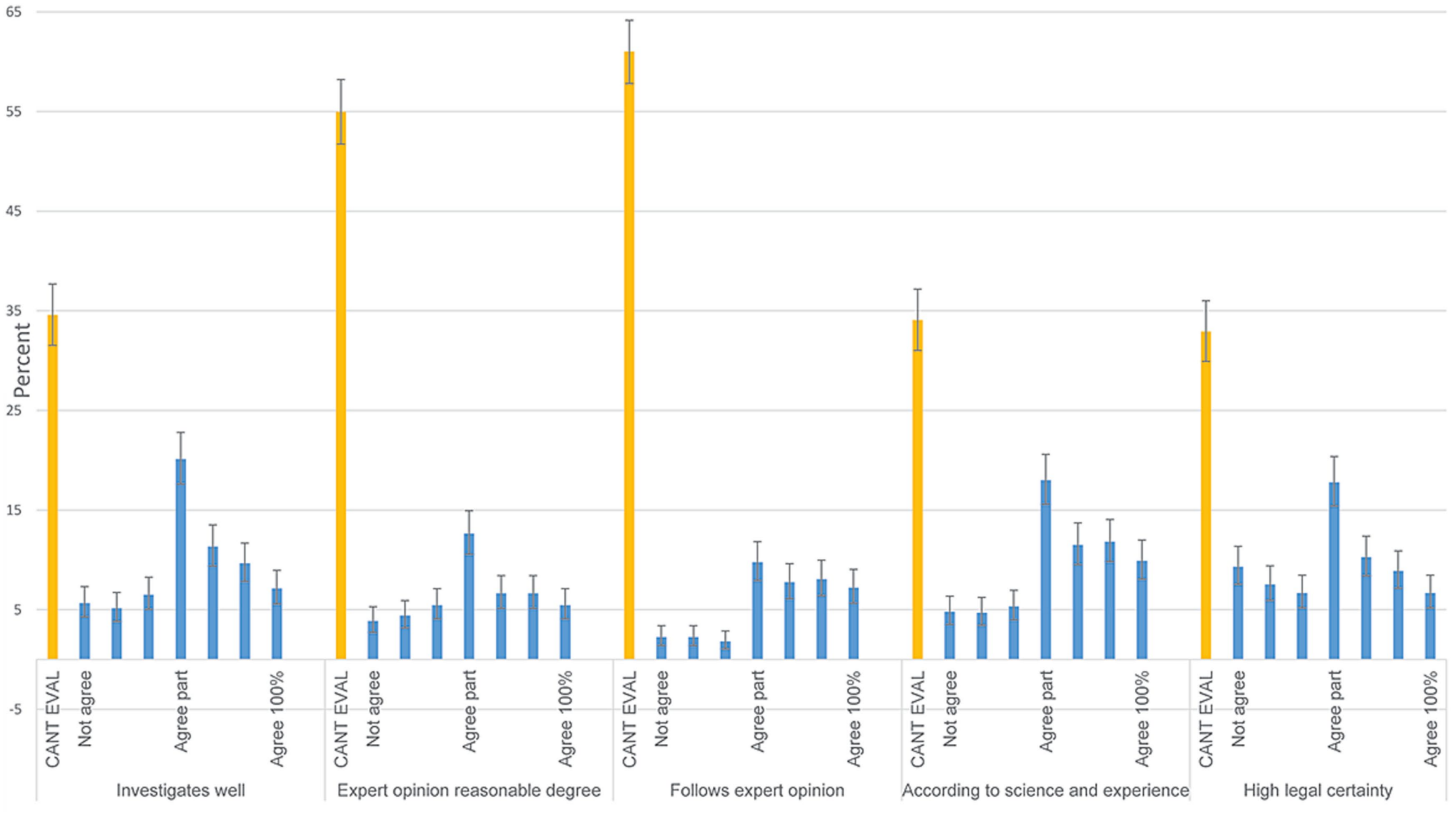
How well do you follow and are acquainted with the work of the Swedish Veterinary Disciplinary Board (VDB). Response distributions to Likert-scale questions from Area 4. The alternatives include cannot evaluate (CANT EVAL) and a 7-step scale from does not agree (not agree) to agrees completely (agree 100%). Denominators from left to right are 955, 935, 944, 940 and 945. 95% confidence intervals are included. Questions in full were posed as: ‘I believe that the VDB investigates cases well’, ‘I believe that the VDB asks for expert opinions to a reasonable degree’, ‘I believe that the VDB follows expert opinions when such have been obtained’, “Are you sometimes surprised that the VDB in one case frees and in others convicts?’ I believe that the decisions from the VDB are associated with high legal certainty’.

Annually, very few respondent did request case verdicts from the VDB (3.2% of 954; 95% CI; 2.2–4.6), while 77.8% ([95% CI; 75.0–80.4] of 950) regularly read about cases in The Swedish Veterinary Journal (26). For the five questions concerning perceptions of the work of the VDB (Figure 2), many respondent answered ‘cannot evaluate’ (33–61%), while the second most common category was the middle category ‘agree partially’, concerning whether cases are well investigated, whether expert opinion is asked for and followed, whether cases are evaluated taking account of science and established experience and whether case verdicts are associated with legal certainty. Stratifying these questions by whether filed or not (Supplementary Table 1 - sheet Filed), the ‘cannot evaluate’ alternative has similar proportions in the group with 1–2 complaints filed, while in the two groups with 3 or more complaints the ‘cannot evaluate’ shares are lower. In the group with >5 complaints the respondents were critical (‘do not agree’ from 53 to 63%).

### Whether verdicts of cases evaluated by the VDB affect your work (area 5)

**Figure 3:**
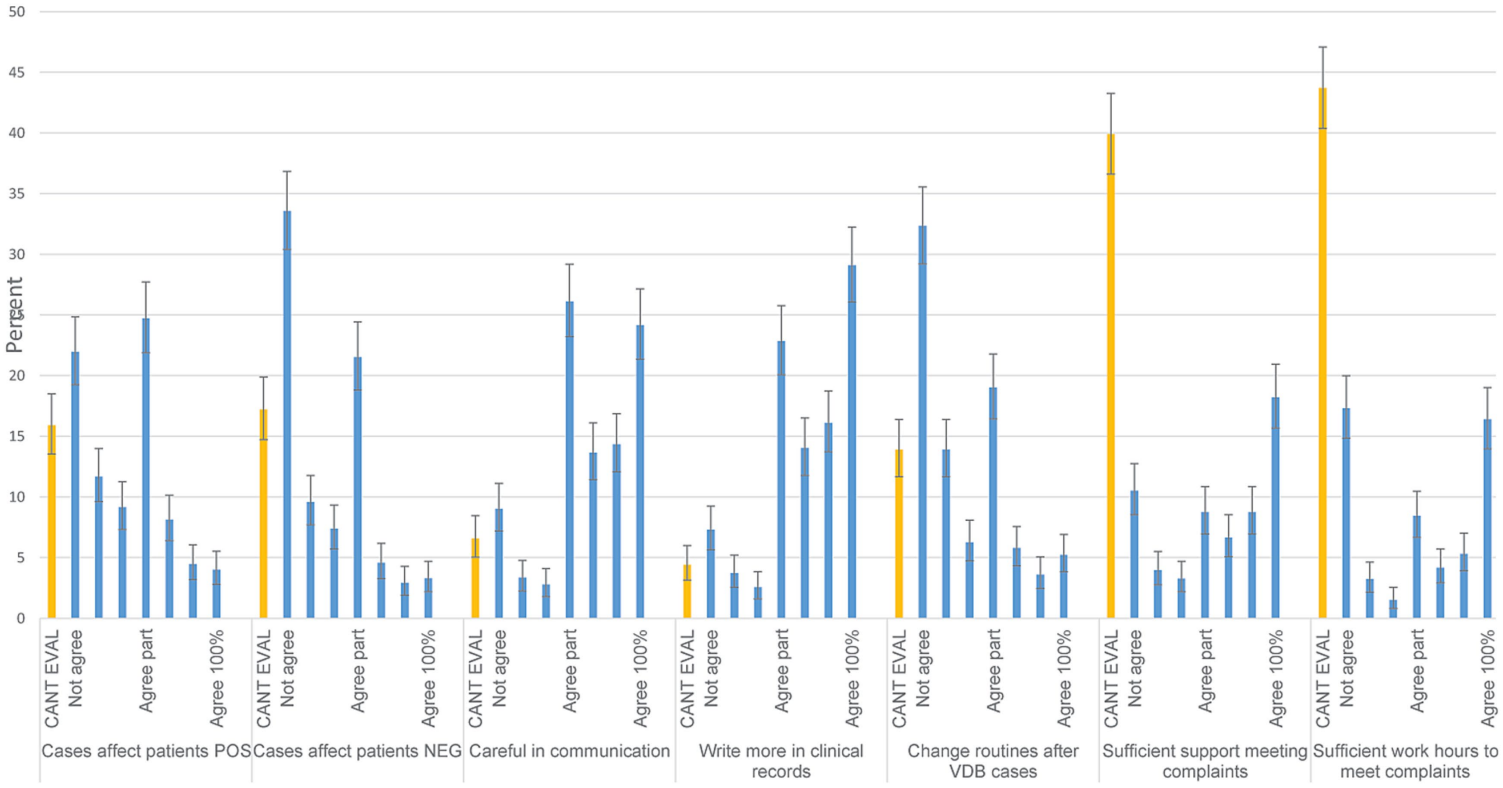
How do assessments from the Swedish Veterinary Disciplinary Board (VDB) affect your work? Response distributions to Likert-scale questions from Area 5. The alternatives include cannot evaluate (CANT EVAL) and a 7-step scale from do not agree (Not agree) to agrees completely (Agree 100%). Denominators from left to right are 874, 855, 865, 863, 863, 857 and 867. 95% confidence intervals are included. Questions in full were posed as: ‘, ‘I believe that the VDB’s positions generally influence how I investigate and treat patients in a way that is positive for the animal’, ‘I believe that the VDB’s positions generally influence how I investigate and treat patients in a way that is negative for the animal’, ‘I believe that the VDB’s positions make me more cautious or careful in my communication with animal owners’, ‘I believe that the VDB’s positions affect how I express myself in writing, how much I write, and how much time I spend on writing clinical records’, ‘At my workplace, we discuss the VDB’s cases and clarify or adjust our routines if we find it appropriate’, ‘At my workplace, sufficient support is provided to respond to a complaint from the VDB’, ‘At my workplace, if a person has complaints filed to the VDB, that person is given time (work hours) to respond to the VDB (i.e., to do so during work hours)’.

The most common response categories for whether the VDB’s positions positively influence the work-up of the patients I admit were ‘partial agreement’ (24.7%, middle category) and ‘does not agree’ (22.0%), and for whether this influence was negative ‘does not agree’ (33.6%) and ‘agree partially’ (21.5%, middle category) (Figure 3). The most common selected alternatives indicate that the VDB has the effects of more careful communication and more emphasis on record keeping (Figure 3). The most common alternative for whether VDB rulings would lead to changes in routines was ‘disagreement’ (32.3%). Most respondents answered ‘cannot evaluate’ to whether support or working time would be provided (39.9 and 43.7% respectively) if a complaint was filed to someone at their workplace. In total 66.7% of the 882 respondents stated they were acquainted with defensive medicine as a concept.

### To admit clients, that either previously submitted complaints to the VDB or an owner with problematic behaviour (area 6)

**Figure 4:**
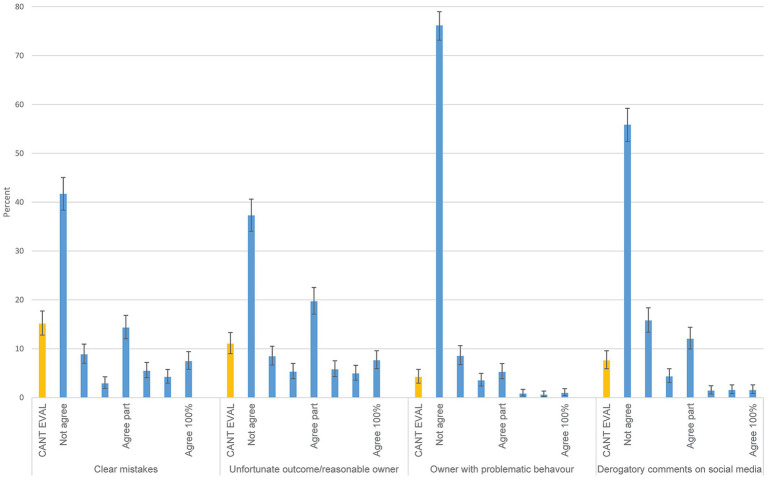
Questions on how animal owners previously filing complaints to the Swedish Veterinary Disciplinary Board or creating other problems would be received at the clinic. Response distributions to Likert-scale questions from Area 6. The alternatives include cannot evaluate (CANT EVAL) and a 7-step scale from do not agree (Not agree) to agrees completely (Agree 100%). The denominators from left to right are 859, 853, 856 and 856. 95% confidence intervals are included. Questions in full were posed as: ‘I would accept owners who have filed complaints concerning me for things I consider to have been clear mistakes, but which did not affect the outcome of the case (perhaps an incorrect note that was corrected, or discharge advice that should have been written but was only given verbally)’, ‘I would accept owners who have filed complaints concerning me for incidents with an unfortunate outcome (e.g., an older dog passed away, but I don’t believe my treatment or advice contributed to this), but where I still think the owner is quite reasonable‘, ‘I would accept owners who I feel have caused significant problems for staff and/or other owners through their behaviour’, ‘I would accept animals from owners who I know have posted ‘cruel’ or derogatory comments, or provided incorrect information, about a named veterinarian on social media’.

To the question whether you yourself would admit an animal owner that had previously submitted complaints 62.8% (95 CI; 59.5–66.0) answered no (*n* = 876), 23.1% (95 CI; 20.3–26.0) do not know and 14.1% (95 CI; 11.9–16.6) yes. The most common answer to whether the clinic would admit the same owner was do not know (39.1% [95% CI; 35.8–42.4]; *n* = 855), yes 33.9% [95% CI; 30–7-37.2]; and no 27.2% [95% CI 24.1–30.1]. Regarding whether clients should be admitted in four specific cases; a client having submitted a claim for simple mistakes, an unfortunate outcome, animal owner with problematic behaviour and animal owner spreading derogatory information it was most common to not agree (41.7, 37.3, 76.2 and 55.8%, respectively, Figure 4). These questions were analysed stratified by number of staff at the clinic, i.e., clinic size (Supplementary Table 1 - sheet ClinSize). Regarding the question concerning whether the respondent would admit an owner previously filing complaints the pattern was similar across categories relating to number of staff. Regarding whether the clinic would admit such animal owners, few clinics (10.1%) with 1–3 staff would admit such animal owners, while 45.8% of the respondents from clinics staffed by >8 stated that the clinic would admit such animal owners (Supplementary Table 1 - sheet ClinSize).

### Complaints filed including sanctioned complaints to the VDB, including support sought and received (area 7a)

In total 524 respondents answered that they had had complaints filed, in 77.9% 1–2 complaints, 18.5% 3–5 and in 3.6% > 5 complaints. From this subset of 524 respondents, 507 answered the question about sanctions; 78.5% were acquitted, 19.1% had had 1–2 sanctioned complaints, 6 respondents 3–5 complaints (1.2%) and 6 respondents over 5 sanctioned complaints (1.2%; Table 2). Using all respondents as denominators, the proportion of men (62.6%) with complaints were larger than for women (47.4%). Even though numerators for those with 3 or more complaints sanctioned were small (*n* = 12), none of these were from female respondents (Supplementary Table 1 - sheet Gender). The proportions with complaints filed increased with years of experience, from 27.8% in those with <1 year of experience to 56.9% in those with >20 years of experience. Similarly, the proportions with sanctioned complaints also did so to some extent, from 0 to 14.4% in the groups with lowest and highest experience (Supplementary Table 1 - sheet Experience).

**Table 2:** Distribution of responses for questions to veterinarians in Sweden, distributed winter 2024/2025, having had complaints filed to the Swedish Veterinary Disciplinary Board (VDB) (area 7a).

Question/category	n	%	95% CI
How many times have you have complaints filed to the VDB? (If you have never been reported, please go to the next page).	524		
1–2	408	77.9	(74.1, 81.3)
3–5	97	18.5	(15.3, 22.1)
>5	19	3.6	(2.2, 05.6)
How many times have you been given a sanction by the VDB—a reprimand or a warning?	507		
0	398	78.5	(74.7, 82.0)
1–2	97	19.1	(15.8, 22.8)
3–5	6	1.2	(0.4, 02.6)
>5	6	1.2	(0.4, 02.6)
I felt that I received support from colleagues during and after the reports.	518		
Do not agree	62	12.0	(9.3, 15.1)
2	34	6.6	(4.6, 09.1)
3	36	6.9	(4.9, 09.5)
4	71	13.7	(10.9, 17.0)
5	69	13.3	(10.5, 16.6)
6	65	12.5	(9.8, 15.7)
Agree completely	181	34.9	(30.8, 39.2)
I felt that I was treated negatively by colleagues during and after the complaints.	513		
Do not agree	433	84.4	(81.0, 87.4)
2	24	4.7	(3.0, 06.9)
3	16	3.1	(1.8, 05.0)
4	10	1.9	(0.9, 03.6)
5	6	1.2	(0.4, 02.5)
6	6	1.2	(0.4, 02.5)
Agree completely	18	3.5	(2.1, 05.5)
I sought support from the Swedish Veterinary Association in connection with the complaints.	521		
Yes	45	8.6	(6.4, 11.4)
No	476	91.4	(88.6, 93.6)
If you sought support from the Swedish Veterinary Association, how useful did you find the information you received?	45		
Not at all	20	44.4	(29.6, 60.0)
2	4	8.9	(2.5, 21.2)
3	1	2.2	(0.1, 11.8)
4	7	15.6	(6.5, 29.5)
5	3	6.7	(1.4, 18.3)
6	1	2.2	(0.1, 11.8)
Very much	9	20.0	(9.6, 34.6)
Did you seek legal assistance in connection with having complaints filed?	520		
Yes	62	11.9	(9.3, 15.0)
No	458	88.1	(85.0, 90.7)
It is my belief that the reason I have been reported to the board on one or more occasions was due to a lack of routines at the workplace—issues that could have been prevented through a different organisation of the workplace (e.g., better facilities, increased staffing, higher competence among the staff, greater influence over the choice of equipment).	517		
Cannot evaluate	28	5.4	(3.6, 07.7)
Do not agree	270	52.2	(47.8, 56.6)
3	35	6.8	(4.8, 09.3)
4	14	2.7	(1.5, 04.5)
5	71	13.7	(10.9, 17.0)
6	25	4.8	(3.2, 07.1)
7	22	4.3	(2.7, 06.4)
Agree completely	52	10.1	(7.6, 13.0)

Presented are numbers in column 2, percentages and 95% confidence intervals (95% CI).

Regarding support from colleagues, only 25% of the respondents thought they got no support or only little support, adding the three first categories (Table 2). Similarly, most respondents (84.4%) disagreed over the statement that that they had been negatively treated by colleagues in conjunction with complaints filed. In conjunction with having complaints filed, 8.8% sought support from the Swedish Veterinary Association. Of these 45 respondents, 20 found ‘no value’ in the provided information. Of 520, 62 had sought legal assistance when having complaints filed. Regarding the question, whether a reason for the complaint filed was shortages at the workplace, that could be improved by introduction of operational oversight, 52.2% answered that they ‘did not agree at all’, while 10.1% ‘agreed fully’.

### Effects on work from having complaints filed (area 7b)

**Figure 5:**
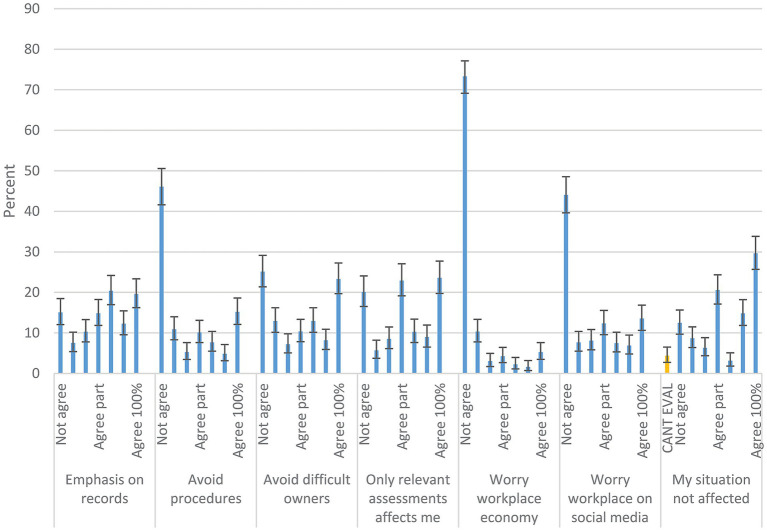
How much your own experiences of having complaints filed to the Swedish Veterinary Disciplinary Board (VDB) have affected your work. Response distributions to Likert-scale questions from Area 7B. How much your own experiences of having complaints filed to the Swedish Veterinary Disciplinary Board (VDB) have affected your work. The alternatives include cannot evaluate (CANT EVAL) and a 7-step scale from do not agree (Not agree) to agrees completely (Agree 100%). Denominators from left to right are 505, 495, 502, 498, 494, 495 and 506. 95% confidence intervals are included. Questions in full were posed as: ‘I devote more time and care to writing clinical records’, ‘I avoid performing certain types of veterinary care procedures’, ‘I avoid ‘difficult’ animal owners as much as possible’, ‘I choose to let my work be influenced only where I believe the VDB has made a good and relevant assessment’, ‘I worry about the workplace’s finances in connection with my case(s) with the VDB’, ‘I worry about the reputation of the workplace (e.g. on social media) in connection with my cases (s) with the VDB’, ‘My situation has been very little affected by the investigations by the VDB’.

Figure 5 demonstrates that some respondents with previous complaints state that they put more emphasis on clinical records, that some avoid difficult animal owners, that some evaluate that only relevant assessments affect them and that their situation has not been affected by complaints filed to the VDB, with a large spread over most response categories for all these four questions. Regarding the other three questions (Figure 5), 46.1% of the respondents stated that they ‘do not agree at all’ with that they avoid veterinary care procedures as a consequence of having complaints filed to the VDB, and 73.3% stated they ‘do not agree at all’ with worrying about workplace economy and 44.0% ‘does not agree at all’ that they would worry about the reputation of the workplace on social media. The respondents with complaint filed answered whether they had had switched to part-time work (it was not asked whether receiving complaints contributed to the decision). This was selected by 132 out of 500 respondents (26.4, 95% CI; 22.6–30.5).

### Perceived importance of reasons for complaints filed to the VDB (area 8)

**Figure 6:**
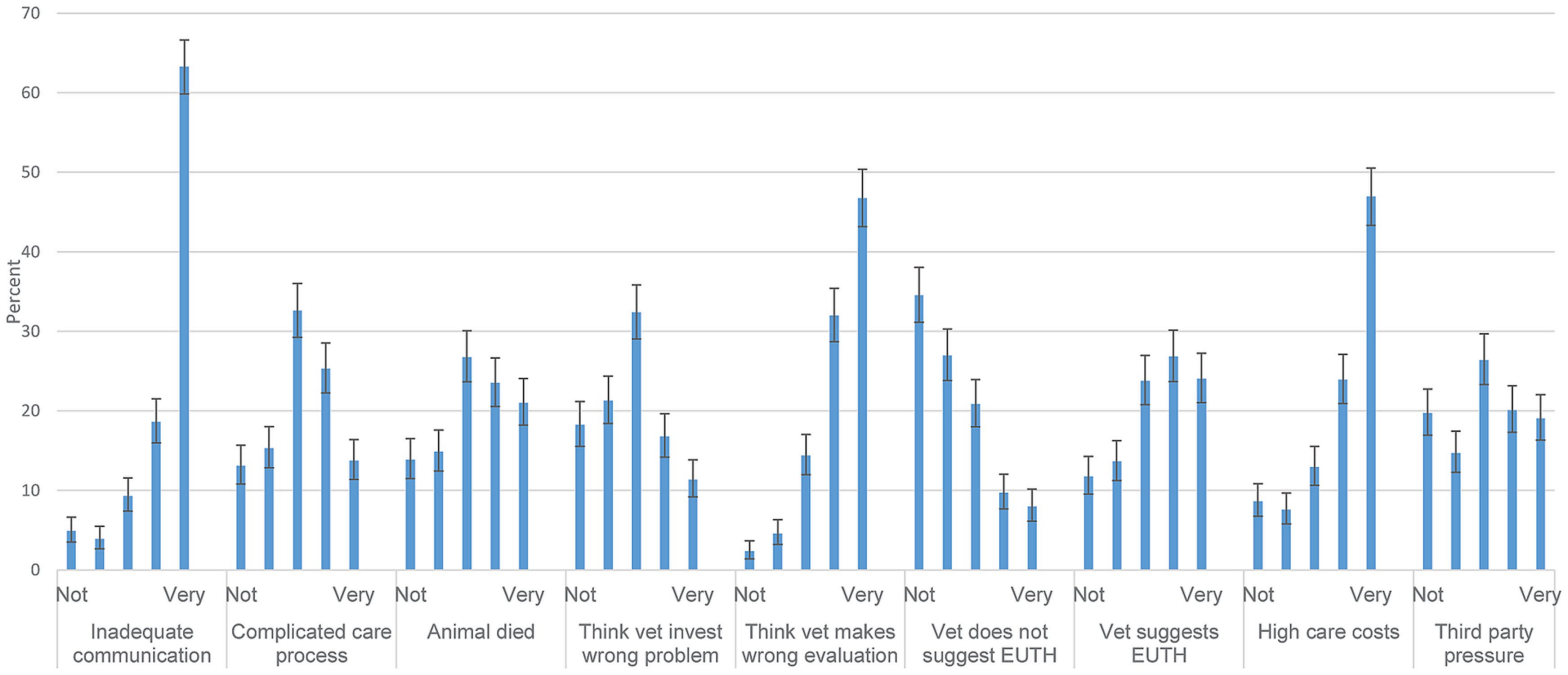
The Swedish Veterinary Disciplinary Board (VDB)- the animal owner, the animal and the veterinary care process. Response distributions to Likert-scale questions from Area 8. The overall question was posed as—Rank the circumstances that you think lead animal owners to file complaints regarding veterinarians to the VDB (1 not at all important, 5 very important). You can, for example, rank multiple factors as not important at all (1). Denominators from left to right are 795, 771, 766, 757, 766, 753, 757, 765 and 761. 95% confidence intervals are included. Questions in full were posed as: ‘Inadequate communication between veterinarian and animal owner’, ‘The veterinary care process became clearly complicated when looking back on it’, ‘The animal died, even though the initial prognosis was assessed as relatively good’, ‘The animal owner feels that the veterinarian is investigating the wrong problem’, ‘The animal owner feels that the veterinarian makes an incorrect assessment or provides incorrect treatment’, ‘The veterinarian does not suggest euthanasia or less costly measures, despite the animal being older and the animal owner wanting to reduce costs’, ‘The veterinarian suggests or advocates for euthanasia, which contradicts the animal owner’s perception of the animal’s health status’, ‘High cost of care—including cost items that the animal owner does not understand’, ‘The animal owner has chosen to report after ‘pressure’ from a third party’.

The most common answer to the question about the influence of inadequate communication was ‘very important’ (63.3%); to whether a complicated care process would lead to complaints- ‘somewhat important’/middle category (32.6%); to whether the animal dies despite a rather positive prognosis at outset ‘rather important’ to ‘very important’ (71.2% combining the three last categories) (Figure 6). Further, that the veterinarian investigates the wrong problem was somewhat less important, while that the animal owner thinks the veterinarian makes incorrect assessments or provides incorrect treatment was considered important with 46.7% answers pointing to ‘very important’. That the veterinarian does not suggests euthanasia in an animal owner-perceived bad condition was not suggested to be a very important reason (‘not at all important’ 34.5%), while the veterinarian suggesting euthanasia in an animal that the animal owner has not realised to be in a bad condition was considered rather important (74.6% combining the last three categories). Even of high care costs are not evaluated by the Swedish VDB, these were still considered important, 43.3% stating this was ‘very important’, while third party pressure was evenly answered over the five categories.

**Figure 7:**
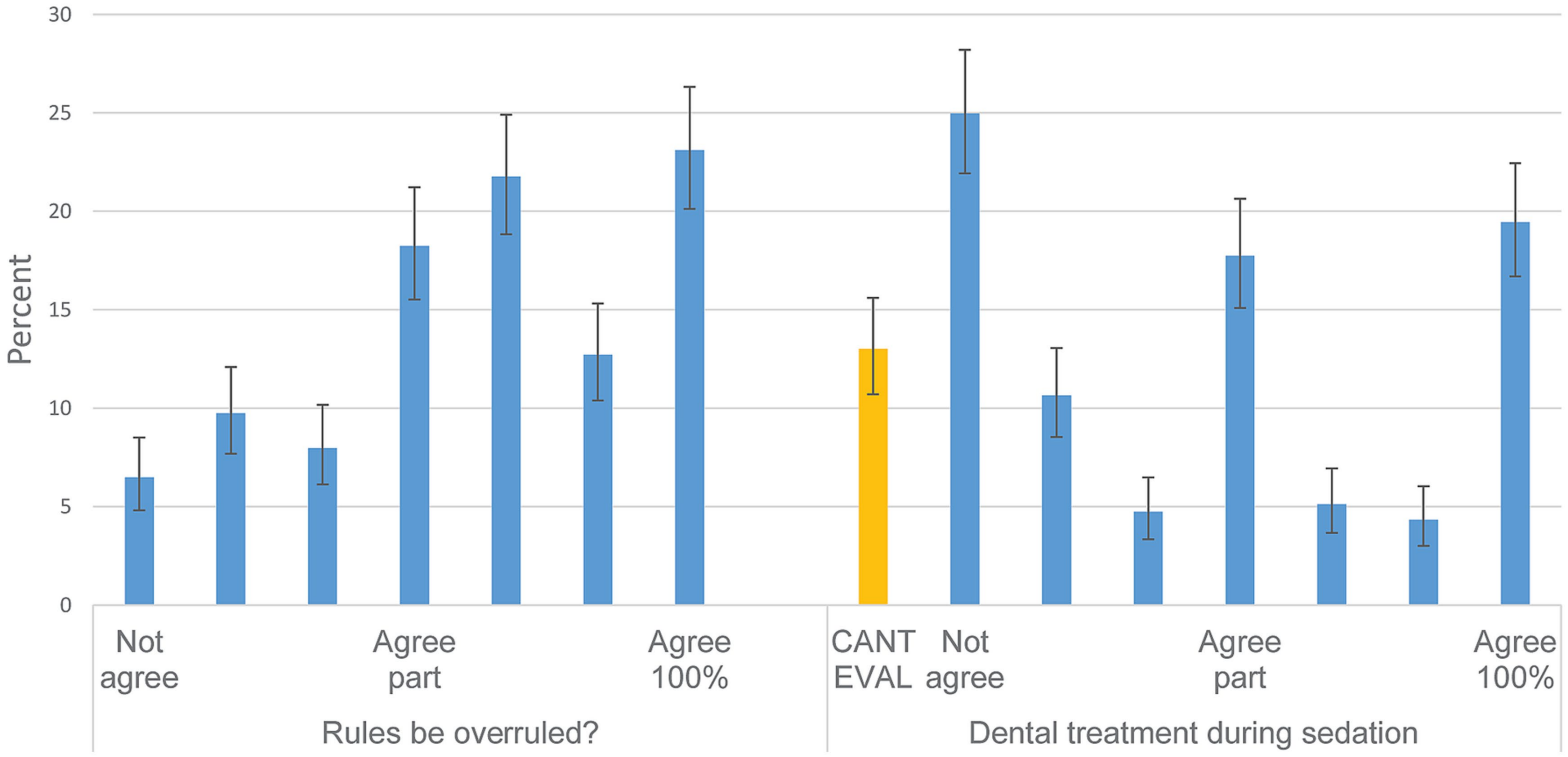
Can guidelines be overruled? Including one example. Response distributions to Likert-scale questions from Area 8. The alternatives include cannot evaluate (CANT EVAL) and a 7-step scale from do not agree (Not agree) to agrees completely (Agree 100%). Denominators from left to right are 740 and 761. 95% confidence intervals are included. Questions in full were posed as: ‘If there are rules and guidelines for how investigation and treatment should be carried out (through legislation, evidence, and proven experience), do you believe that one should be able to deviate from these without being found guilty by the VDB, based on the animal owner’s wishes and financial circumstances, provided that this is justified in the clinical record?’, ‘With this suggestion in mind, what do you think about the following example (based on the Swedish Veterinary Association’s guidelines)?” “I believe that a veterinarian who, in consultation with the animal owner, chooses to sedate rather than anesthetize a dog or cat for dental treatment should not be immediately found guilty by the VDB because of this’.

There were two questions about whether rules or guidelines could be overruled to some extent, to provide health care to animal owners who wanted for example cheaper alternatives, to which many agreed (Figure 7). Combining ‘partial agreement’ to ‘complete agreement’ (the four last categories), 75.8% agreed that this was the case, while for the practical example, whether dental treatment could be performed during sedation, respondents were more cautious with 25.0% directly disagreeing.

### Views about an ‘ideal’ VDB (area 10)

**Figure 8:**
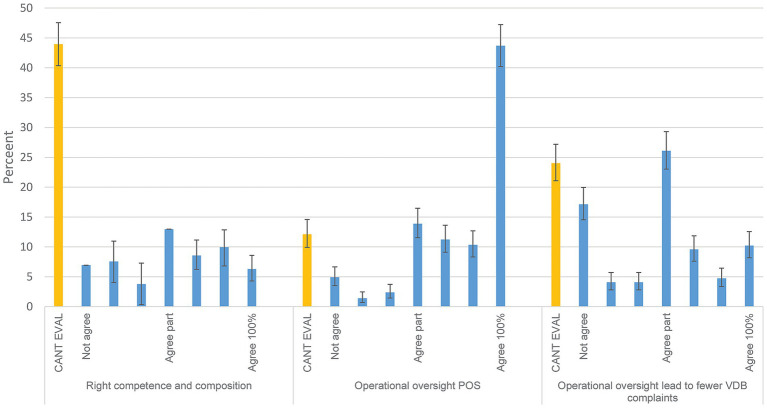
The ideal Veterinary Disciplinary Board (VDB)? Operational oversight etc. Response distributions to Likert-scale questions from Area 10. The alternatives include cannot evaluate (CANT EVAL) and a 7-step scale from do not agree (Not agree) to agrees completely (Agree 100%). Denominators from left to right are 794, 792 and 782. 95% confidence intervals are included. Questions in full were posed as: ‘It is my opinion that the VDB has the right competence and composition’, ‘I believe that the introduction of operational oversight is positive as a complement to individual oversight for animals that need veterinary care in Sweden—meaning that quality and accessibility are improved’, ‘To what extent do you believe that operational oversight will lead to fewer complaints being made against veterinarians to the VDB regarding animal health and veterinary care?’.

To the question whether other persons than the animal owner or the caretaker should be able to file complaints to the VDB, 63.6% (95 CI; 60.1–66.9) thought not, 18.5% (95 CI; 15.9–21.4) answered do not know and 17.9% (95% CI; 15.3–20-7) stated yes (*n* = 799). The responses (*n* = 783) to whether there should be a fee associated with filing a complaint were yes 56.6% (95 %CI; 53.0–60.1), no 26.2% (95% CI; 23.1–29.4) and do not know 17.2% (95% CI; 14.7–20.1). To the question whether you experienced, or heard others refer to, that complaints filed to the VDB have occurred in association with complaints filed to the County Administrative Board by the veterinarian due to inferior animal welfare; of 769 answers equal shares said no (50.2%) and yes (49.8%). Further, to the question whether you heard about cases of extorsion of a veterinarian, where the animal owner threatening to scandalise through social media or to file a complaint to the VDB, 61.5% (95% CI; 57.9–64.9) stated yes and 38.5% no (95% CI; 25.1–42.1). Figure 8 shows that most respondents thought they could not evaluate the composition and construction of the VDB (42%). Further many respondents agreed that an operational oversight would have positive effects on quality and access to veterinary care, 43.7% ‘agreed completely’. However, to the question whether an operational oversight would lead to fewer complaints to the VDB, the most common answer was ‘partial agreement’ (26.1%). To the question concerning whether an animal owner asks for a clinical record; would this in some cases cause hesitation or be denied, 76.7% (95% CI; 73.6–79.6) said no and 13.5% yes (95% CI; 11.2–16.1). The last question whether the respondent would recommend an animal owner, concerned about the costs of a certain case, to file an errand to the National Board for Consumer Disputes (27) 51.5% (95% CI; 48.0–55.1) said yes and 17.8% no (95% CI; 15.1–20.6). The last group 30.8% (95% CI; 27.5–34.1) were unaware that ARN could handle veterinary care costs complaints.

## Discussion

### The sample and the population

The targeted group was veterinarians in Sweden working clinically (28) or having worked clinically, around 2,800 veterinarians. The 1,000 veterinarians opening the link to the questionnaire suggests we reached more than one third of the target population (1,054/2,800). This is likely a larger share compared to many other studies on the same subject. Over 80% of the respondents were female, similar to the national gender distribution of veterinarians with 74% women (29). Over 67% of the respondents had more than 11 years of experience of clinical work. Taken together this implies that the study population to a large degree represents practitioners with large experience from the field. An overweight of females, mainly working in companion animal practice were found to be similar to respondent distributions from questionnaire samples addressing suicidal thoughts in the Norwegian veterinary population (30) or experiences of the Dutch VDB (3). Actually, very few complaints (0.4%) filed relates to production animals and 12% to horses (7).

Almost 50% of the respondents worked in smaller enterprises, a quarter owning or co-owning their practice. This share of owner or co-ownership was similar to that of the Norwegian veterinary population (30), but less than in the Dutch veterinary population (3). In our sample, 17% had more or less left clinical practice. However, few of these stated that the VDB or the regulatory organisation had a large impact on this decision. In the Dutch study (3) only 3% had left clinical practice.

In this discussion we do both national and international comparisons, guided by the idea that client complaints committees may be more optimal in some countries than other. However, there are more to what is best for a society, e.g., regarding between-country differences in the veterinary sector, than what is possible to evaluate here.

### Respondents with complaints—with and without sanction

Using all answers (*n* = 1,054) producing conservative estimates given that not all answered the questionnaire in full, about half of the respondents (50%) had at least one complaint filed to the VDB. This result is considered representative considering that there were 2,816 clinically active veterinarians in Sweden per January 2025 (28) and a yearly average of 200 complaints being filed with the VDB (7). There may be a slight overrepresentation of veterinarians who received a sanction in this study (21%). This figure can be compared to a recent national average in Sweden regarding complaints filed, where 13% of complaints led to a sanction (7).

Regarding gender, more men (62.6% of all male respondents) than women (47.4%) had complaints filed against them. Part of this association is likely explained by male respondents having more years in the profession compared to female respondents. However, another study from Sweden showed that complaints filed to male veterinarians were associated with sanctions more often than those to female veterinarians, despite men being a minority in the profession (7). In addition, no female respondent reported receiving more than 1–2 sanctions, unlike men. When comparing shares of men and women in the veterinary population in New Zealand, men had more commonly received complaints compared to women (6).

Associations between demographic variables, e.g., experience and workplace, may have confounded overall results. For example, working in clinics owned by corporations was associated with having more complaints filed but also with being more experienced (Supplementary Table 1 - sheet ClinOwnVsExp). It is likely that more years of clinical work and patients admitted increases the likelihood of being reported. In the Netherlands 42% of veterinarians had faced a complaint to the VDB (3). A smaller study from the US (*n* = 92) showed that the rate of complaints to veterinary internists was higher (2). In total 64% had received one complaint during the previous 6 months and 93.5% stated they commonly receive a complaint to the VDB every few months (2). However, in our study, as in most other studies on the same topic, there was a risk of interest bias, as respondents with prior experience of complaints filed with the VBD may have been more, or perhaps less, inclined to complete the questionnaire.

### Work health in general and effects from having complaints filed

Norwegian veterinarians reported on high emotional demands, negative work/life balance and fear of complaints (22). On a scale from 1 (no stress at all) to 5 (extreme stress) veterinarians answered with means from 2.0–3.1, indicating a moderate general stress level (22). We asked whether respondents enjoyed work, combining alternatives 1–3 on a scale of 7, only 10.2% in our study said they did not enjoy work and combining alternatives similarly 25.2% felt they did not have influence over questions of interest at their work. Naturally, respondents working in smaller business more often felt they had large influence (Supplementary Table 1 - sheet Workplace). In the 19 respondents that had had complaints filed >5 times, 10 did not enjoy their work, otherwise the pattern was similar across those with complaints filed and the overall population. We further asked whether respondents feared complaints by the VDB, of which 40% did, combining alternatives 5–7 on a scale of 7 in Figure 1. Earlier work has suggested veterinarians working clinically are indeed affected by VDBs, both when having complaints filed (2, 31) and more in general when deciding on treatment of patients during clinical work (9). Summarizing, our results suggest that complaints to the VDB indeed had an effect on a large minority of the respondents. However, respondents still enjoyed work, indicating that Swedish veterinary population may perhaps be less affected than the other populations studied.

All respondents were asked whether the workplace would provide support and time to address complaints, which many did not know. Stratifying the question by those with complaints filed and those without, the patterns were similar (Figure 3; Supplementary Table 1 - sheet Filed). Most respondents felt supported by colleagues, and few found themselves negatively received by other colleagues (Table 2). This is in accordance with veterinary internists from the USA (2), where 42% strongly agreed with feeling support from colleagues. Feeling support is likely important for coping with the stressful process of receiving a complaint.

Interestingly, regarding effects on the work situation in the population with complaints filed, most response categories, i.e., from no effect to large effect, were evenly selected (Figure 5). However, a third agreed completely with the statement that their situation is not affected at all. For three questions complaints seemed to have some impact: avoiding procedures, worrying about workplace economy, and worrying about the spread of information on social media. It can be concluded that the respondents with complaints filed, as a group, do not consider themselves heavily affected by complaints filed. Even so, 15% of respondents with complaints filed, Figure 5 fully agreed that they avoided similar tasks due to the complaint. This is in agreement with US veterinary internists (2), where 12% reported sometimes avoiding tasks (including both those with complaints and a few without). In a qualitative study, interviewing veterinarians with previous complaints (9), improvement of clinical records and avoiding tasks were addressed, and also avoiding animal owners perceived difficult to manage. We conclude that VDB rulings may sometimes affect the possibility for an animal owner to get help, perhaps in an urgent situation.

### Perceptions and experiences of the process at the VDB

Comparing the answers in the total population to those with previous complaints filed (Supplementary Table 1 - sheet filed), it was the group with more than five complaints filed that disagreed with that expert opinion is used and followed, that the VDB rules according to science and established experience, and with that rulings are associated with high legal certainty. This suggests that veterinarians become increasingly critical the more complaints they get and perhaps the more they receive sanctions. In general, it is likely that persons driving long-standing issues with authorities become increasingly critical, while those without this experience will remain more neutral or positive.

US veterinary internist were asked to evaluate the statements, ‘Overall I believe complaints are reasonably dealt with’, and ‘Overall I believe the complaint process is handled competently’ (2). Most respondents (84 and 86% respectively, (2)) agreed or were neutral, while 65 to 84% (median across questions 74%) agreed of were neutral in our study (Figure 2). With lack of a ‘cannot evaluate’ option (2), comparisons to our results becomes complicated, but the comparison may perhaps suggest that the 92 veterinary internists regarded their VDB more highly. One area not addressed in the current study but cited as a stressing factor in other studies is the duration of processing complaints filed (9). In Sweden the process often takes about a year, a rather long time but this may be needed to achieve legal certainty and efficiency of the VDB (11), where group evaluation of complaints is conducted a few times each year.

### Is the VDB perceived to promote best practice?

Documents held by the VDB are, in general, public under the principle of public access to official documents in the Freedom of the Press Act (32). The identities of both reporting and reported persons are therefore normally public information (and therefore no information asked for in the questionnaire was considered sensitive). However, certain details may be subject to secrecy, for example if disclosure could be assumed to endanger the complainant or a related person (33). However, in the Netherlands there was an overweight for agreement that the VDB promotes best practice and more than half of the respondents also thought that practitioners can learn from the VDB rulings (3). Regarding the question to Swedish respondents thought on how they learn about, and perhaps learn from completed assessment, our study shows that it was only the respondents with frequent claims filed that also frequently ask the VDB for complete assessments (11 out of 19 Supplementary Table 1 - sheet filed). By contrast, 78% of the respondents read about the outcome of complaints in the Swedish Veterinary Journal and this share was similar for those with complaints (Supplementary Table 1 -sheet filed). This suggests that veterinarians are interested and have potential for both general improvement and updating, as well as self-preparation regarding possible complaints and the work of the VDB. Reading the complete information about cases has potential for increased knowledge of the process at the VDB compared to reading just extracts. Increased focus from practitioners on the process at the VDB, and to generally prepare for dealing with complaints, both practically and mentally (9) may be a way to make complaints handling less frustrating, perhaps extending to both basic and continuing education of veterinarians. Perhaps this could contribute to increased transparency of the work of the VDB.

### Is the VDB perceived to promote contextualised care?

In some contrast to gold standard care this study also explored perceptions on if care could be adapted for animal owners with smaller budgets (Figure 7) and asked about awareness of defensive medicine, known to raise human healthcare costs (19). Regarding a hypothetical question about if performing veterinary dentistry under sedation only, contrary to national and international recommendation that advocate general anaesthesia (34) should render a sanction from the VDB- veterinarians were of different opinions. Although dentistry is recommended to be performed under general anaesthesia (34), it is common to perform procedures under sedation only (35) which may be a more affordable alternative (20, 21), more recently addressed as contextualised care (22, 36).

Awareness of the concept of defensive medicine was high (67%) and consistent across gender and experience (Supplementary Table 1 - sheets Overall and Gender). Answers were ranging from ‘Agrees completely’ to ‘Do not agree at all’. Our findings indicate that many veterinarians are open to providing care that does not always follow the gold standard, depending on the context. This result is in line with the practice of spectrum of care medicine.

### General effects of the VDB on clinical practice and reasons for complaints filed

Many respondents agreed that the VDB leads to carefulness in communication with animal owners (52% combining the responses 5–7 on a scale of 7, Figure 3), and more emphasis on clinical records, but few agree that routines are changed after VDB assessments. The pattern for these questions was similar when stratified by years of experience. This is in line with research from California suggests that improved record keeping can be a deciding effect for avoiding sanctions by the VDB (5). This also agrees with a thematic study from the UK (9), where improved record keeping and carefulness in communication were addressed as consequences of having complaints filed to the VDB. US veterinary internists evaluated the statement ‘I feel the need to please my clients to avoid complaints against me’ and 63% strongly or partially agreed (2). In the current study, ineffective/poor communication was also an important perceived reason for complaints filed (Figure 6). Communication gaps have been addressed as a major point in many previous studies (1, 3, 6, 9). Professional behaviour is another common determinant of studies addressing reasons for complaints filed, also in veterinary medicine (e.g., (6, 9)). These factors highlight the importance of education and knowledge in so-called soft skills (7, 37) complementing medical skills.

As in the current study, high care costs (Figure 6) have also in other studies been addressed as cause of complaints (2, 6, 9), and health care costs have indeed increased (17, 38, 39). There is a risk that a high care cost contributes to many complaints filed, also when cost of care is not evaluated in itself. In Sweden, cases of perceived veterinary overcharging are currently addressed by the National Board for Consumer Disputes (27). ARN examines disputes between consumers and businesses, including veterinary fee disputes. ARN’s decisions are recommendations and not legally binding but are often complied with in practice. Proceedings are free of charge for the consumer and may be an alternative to filing a complaint with the VDB in cases concerning price or reimbursement. In this study, 52% of respondents would refer cost-related complaints to ARN; however, 31% were unaware of its role, indicating potential for improvement in directing dissatisfied animal owners to ARN instead of the VDB. Also, few of the respondents in our study thought that expanding the scope of the VDB to address overcharging was a good idea.

### Admitting animal owners that have filed complaints

Most respondents would not like to admit animal owners that have filed complaints, or disrupted the work at the clinic, e.g., by being rude (40), or who have spread derogatory information on social media. Some answered that the clinic, but not themselves, may admit animal owners that made complaints previously. This was especially true for larger clinics (Supplementary Table 1 - sheet ClinSize). More possibilities for colleague support in larger clinics seems a plausible explanation for this finding. Overall, these findings suggest that some animal owners may risk having problems receiving care for their animal, if the veterinarians are aware of previously filed complaints. In Sweden there is no legislation that states that privately practicing veterinarians must admit owners. However, government-employed veterinarians, e.g., district veterinary officers (25) have a responsibility to provide care for animals 24–7 (41).

### Threats of extorsion or slander

Around half of respondents had heard of VDB complaints that were preceded by reports of an animal owner to the County Administrative Board for inadequate animal husbandry. In Sweden such reactive filing was found in 4 of 47 euthanasia-related complaints (8). While the possibility that different forms of retribution may cause concerns among veterinarians, the extent is unclear, particularly in the era of social media where slander has become facilitated. Most US veterinary internists agreed with the statement that ‘there is no recourse against unfair online reviews’ (2). In the same study (2), over 50% reported knowing someone who had reimbursed an animal owner to avoid a complaint or negative post. Given the prolonged nature of complaint handling, perhaps especially in privately owned clinics such solutions may be seen as pragmatic. In the UK, mediation services (42) may after mediation with both parties suggest financial settlements between parties as part of complaint resolution. Summing up, it seems that reimbursements of animal owners when owners perceive that outcome and cost does not match are more openly promoted in some countries, e.g., in UK (42) or in Denmark (43) where the VDB can advise monetary reimbursement. In Sweden, ‘officially’ such reimbursement is done if advised by ARN (27) but could occur more ‘unofficially’ at some clinics to prevent, e.g., complaints to the VDB.

### Organisation of the VDB and operational oversight

Over half of the respondents considered it a good idea to add a fee for the complainant when filing a complaint (strongly suggested by (3)), while few thought that additional persons other than currently (only animal owner, caretaker and the Administrative County Board) should be able to file complaints. Interestingly, for veterinarians to file complaints against themselves or colleagues is an option in, e.g., Norway (44) and it is also reported from the Netherlands (1, 45). Given that evaluations would be perceived as being associated with a high degree of legal certainty, filing a complaint against oneself may be an option for practitioners wanting to have a specific type of case tested or to simply file a complaint if the animal owner is concerned.

Regarding operational oversight, it is supported by the respondent population. In total 70% (response options 5–7 on a scale of 7, Figure 8) are generally positive to this introduction, while rather few think that it can alleviate the current level of complaints. Operational oversight may operate in two ways; complaints that after initial evaluation are found to relate to organizational matters could be directly forwarded. Operational oversight could also take the form of external auditing. External auditing can be combined with internal efforts. It is unknown to what degree in-house quality assurance processes are used, even if it is more likely that larger organisations (46), compared to single-veterinarian practices, put more emphasis on formal systematic quality control based on that they have more persons to coordinate.

### Comparison of organisations of disciplinary boards in different countries

A recent study describes that structures of medical disciplinary boards vary widely across the world (47). Also, VDB structures vary across countries (4). For example, Norway charges a fee to file complaints (44), unlike Sweden and the UK. The UK often resolves complaints through mediation (42) before reaching the VDB (48), a system not found in Norway or Sweden. Sweden (11) publicly discloses personal details from complaints (upon request), Norway anonymizes data online (40), and the UK names only the veterinarian and only if complaints reach disciplinary hearings (48). Public exposure in the UK is a source of stress (9). In recent years, Sweden handled over 200 complaints (49)—about three times more per capita than Norway (~30/year), and more than the UK’s ~ 20 published cases. Committees also differ: some issue sanctions or can revoke license, e.g., Sweden (12) and UK (50), others are advisory (44), or suggest sanctions or reimbursement (5, 43) or combinations thereof. While US vets report high complaint loads alongside general acceptance of the VDB’s role (2), it could be speculated that veterinarians can adapt to receiving many complaints. However, Swedish vets facing frequent complaints report significant stress, highlighting the need for support and transparency during the process (Figure 5). Our suggestion is that regulators should, when designing or redesigning VDBs as has recently been done in Sweden, inform themselves of functions and effects of disciplinary boards internationally.

### Limitations

There were some limitations in this exploratory cross-sectional study. The survey was anonymous and not systematically randomised. Therefore, we could not examine which segments of the veterinary population answered the questions. This may have created selection bias. Neither could we verify if an individual answered the questionnaire multiple times. We posed many questions and through the 95% CIs presented a large number of pairwise comparisons can be made. Surveys are inherently subject to biases, such as question/response wording bias, acquiescence bias, and recall bias (51). Non-response bias occurs when those who choose not to respond differ systematically from those who do—for example, individuals more dissatisfied with the VDB may have responded at a higher rate than those who are satisfied or indifferent. To minimize risk of drop-out due to this we included the response alternative ‘cannot evaluate’, which proved useful. It is however important to note that the majority of the studies we have compared our results to have similar issues with, e.g., non-response bias and recall bias. While comparing study results to results from other studies is key when discussing results, comparing between studies with unknown degrees of bias may yield biased conclusions. Although we aimed for international comparisons in this study, no collaborators were found abroad. The free-text answers warrant scrutiny and addressing, together with the quantitative material, but could because the presentation is already long not be included.

### Conclusion

While VDBs are considered important parts in animal healthcare, in Sweden many veterinarians, but far from all, fear being reported. The high number of respondents indicate the importance of studying the effects of the VDB and being reported to the VDB. Many veterinarians are relatively unfamiliar with processes at the VDB and relatively few had received sanctions. Education in soft skills and preparedness for complaints may therefor add value. A large share of the respondents thought that not only gold standard care should be sufficient in certain circumstances, suggesting the VDB should have less emphasis on standard of care being “hospital oriented,” instead recognising and allowing the range of care of smaller clinics. To ensure legal certainty and predictability, and to minimize stress for those having had complaints filed against them, it is important that the processes are transparent, and evaluations are evidence-based, thus following science and proven experience.

## Data Availability

Some fields are removed to make anonymization complete, the rest of the data are available from the corresponding author. Requests to access the datasets should be directed to agneta.egenvall@slu.se.
